# Imaging of cerebral complications of extracorporeal membrane oxygenation in infants with congenital heart disease — ultrasound with multimodality correlation

**DOI:** 10.1007/s00247-019-04603-1

**Published:** 2020-02-15

**Authors:** Patricia Svrckova, Riwa Meshaka, Melanie Holtrup, Angela Aramburo, Kshitij Mankad, Farhat Kazmi, Catherine M. Owens, Sandra Gala-Peralta, Thomas Semple

**Affiliations:** 1 Radiology Department, The Royal Brompton Hospital, Sydney Street, London, SW3 6NP UK; 2 Imaging Department, University College Hospital, London, UK; 3 Paediatric Intensive Care Unit, The Royal Brompton Hospital, London, UK; 4 Great Ormond Street Hospital, London, UK; 5 National Heart and Lung Institute, Imperial College London, London, UK

**Keywords:** Brain, Complications, Congenital heart disease, Extracorporeal membrane oxygenation, Neonate, Ultrasound, Vascular imaging

## Abstract

Cranial ultrasound on neonatal intensive care units is generally performed by intensive care physicians, but radiologists often provide this crucial bedside test to children on specialist paediatric cardiac intensive care units. On a paediatric cardiac intensive care unit, complex congenital cardiac conditions are commonly encountered in both pre- and postoperative scenarios, often with the use of extracorporeal membrane oxygenation (ECMO), which both increases the risks of a number of neurologic complications and results in significant changes in vascular physiology. The aim of this pictorial essay is to discuss cranial ultrasound technique, demonstrate the changes in Doppler flow profiles resulting from veno-arterial extracorporeal membrane oxygenation and congenital cardiac conditions, and illustrate commonly encountered intracranial complications of extracorporeal membrane oxygenation support in congenital cardiac care.

## Introduction

In the United Kingdom, most cranial ultrasound (US) examinations are performed in the context of prematurity, by neonatal intensive care physicians. However, in specialist paediatric cardiac surgical centres, radiologists often provide this crucial bedside imaging service. In these units, veno-arterial extracorporeal membrane oxygenation (ECMO) is commonly used as a form of periprocedural support in neonates with particularly complex congenital heart diseases.

These children sometimes sustain significant neurologic complications, both from their underlying cardiac disease and subsequent surgical intervention, as well as from anticoagulation and the ECMO support itself. A two-decade review of neurologic events related to ECMO in The Netherlands reported cranial ultrasound abnormalities in 17% of patients, most frequently haemorrhage (8.8%) and ischaemic stroke (5%), with higher frequencies of complications in veno-arterial ECMO and an interesting predominance of left-sided pathology [1].

Extracorporeal membrane oxygenation support is not compatible with MRI, and at times safe transfer to the CT scanner is not possible. Outside of the few centres employing mobile CT units, this leaves ultrasound as the only immediately available modality. The aim of this pictorial review was to illustrate the US appearance of common and less common intracranial pathologies encountered in neonates and infants with congenital heart diseases, before, during and after receiving ECMO support.

## Extracorporeal membrane oxygenation

**Fig. 1:**
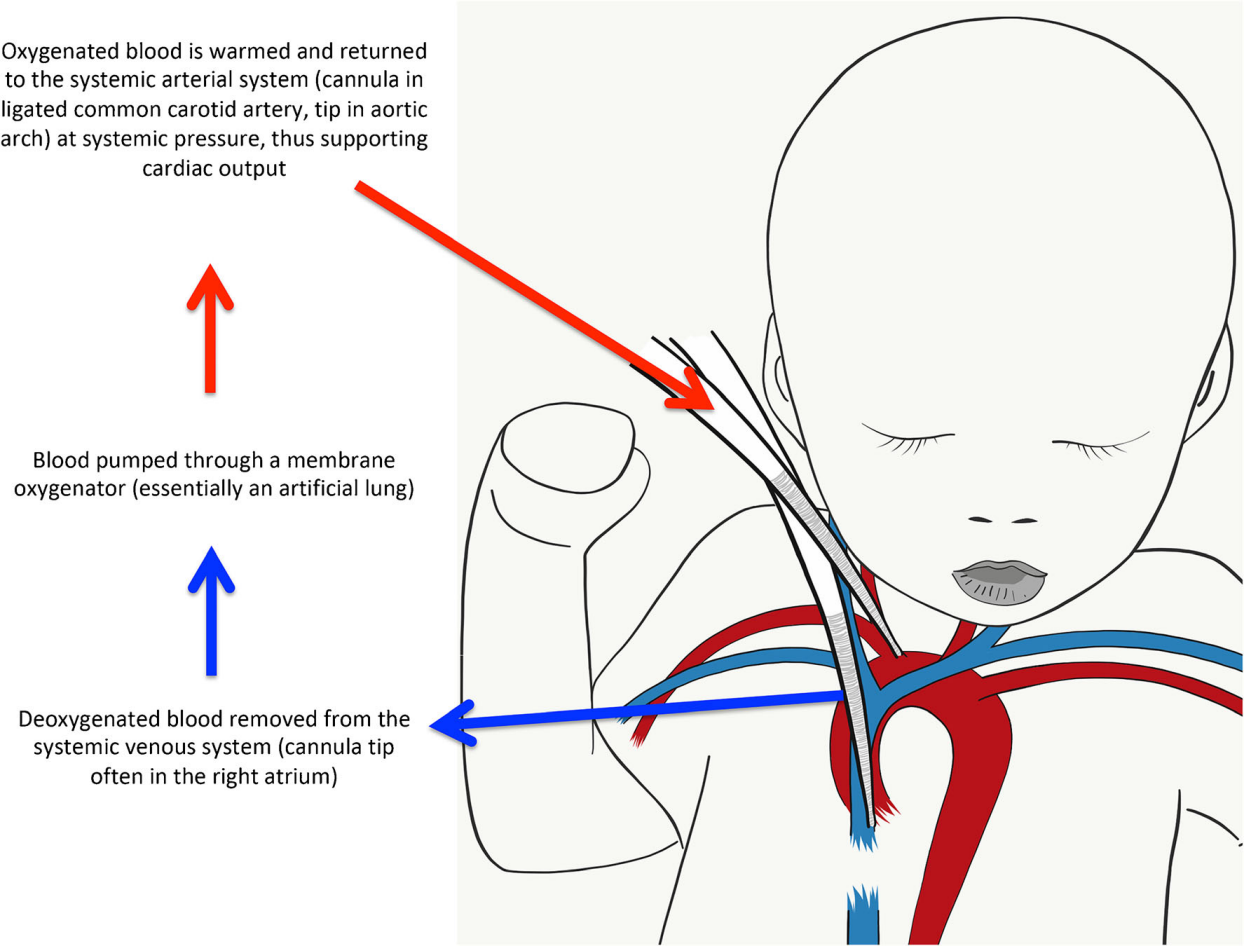
Diagram of veno-arterial extracorporeal membrane oxygenation. There are many more complex extracorporeal membrane oxygenation setups, but this is the most commonly used, with so-called peripheral cannulation of the right common carotid artery and the right internal jugular artery. The right common carotid artery is ligated superior to the cannulation site with right anterior cerebral circulation dependent on the circle of Willis. Drawing by Katriina Nichols

**Fig. 2:**
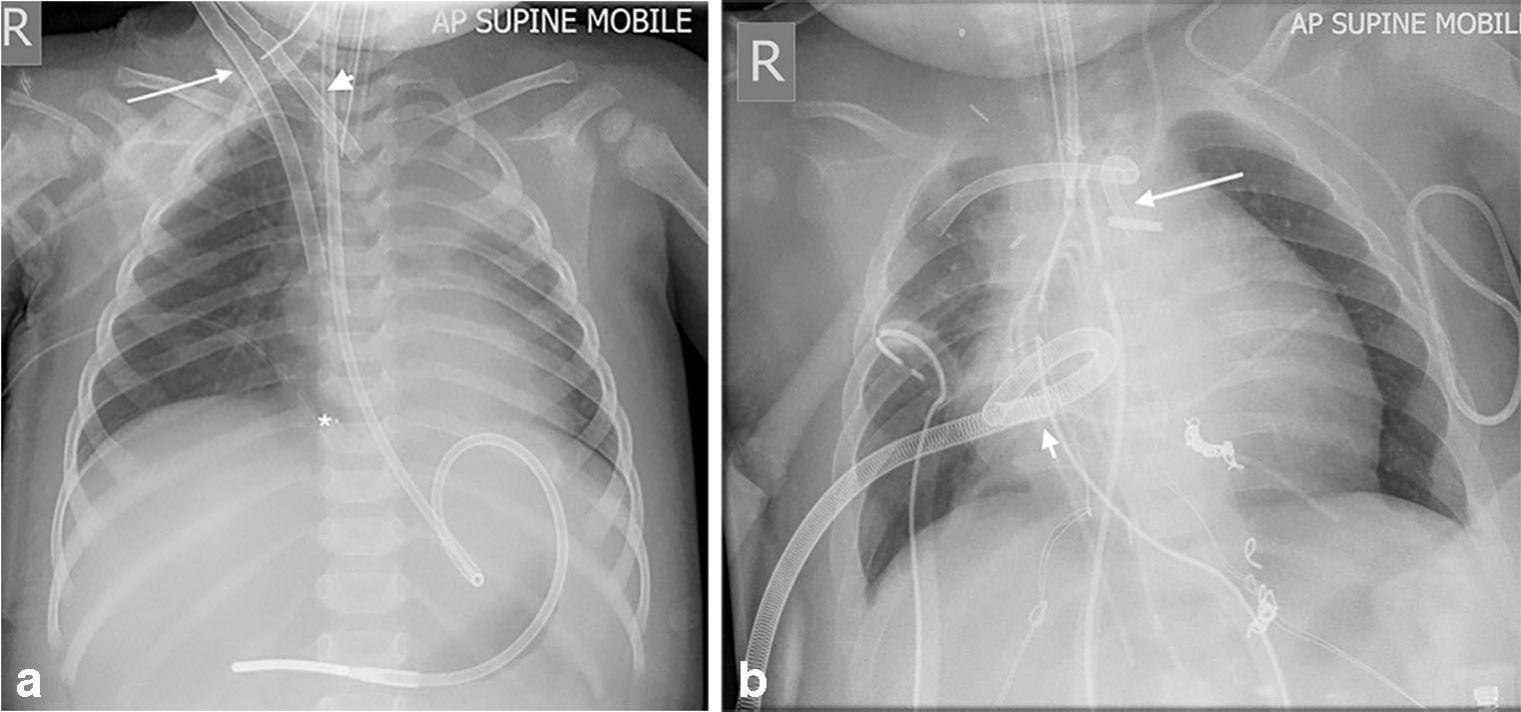
Anteroposterior radiographs demonstrating veno-arterial extracorporeal membrane oxygenation cannula positions. **a, b** Radiographs demonstrate peripheral (**a**) and central (**b**) veno-arterial cannula positions. In peripheral cannulation in a 3-month-old girl (**a**), the venous drainage cannula (*long arrow*) traverses the right atrium with a radiopaque marker at its distal tip (*). The arterial return cannula (*short arrow*) is inserted via a ligated right common carotid artery and has a radiolucent tip in the aortic arch. In central cannulation in a 3-month-old boy (**b**), the venous drainage cannula (*short arrow*) in the right atrium and arterial return cannula (*long arrow*) in the aorta are inserted through a sternotomy at the time of congenital cardiac surgery

In veno-arterial ECMO, blood is diverted from the child’s systemic venous system through an external membrane for gaseous exchange, and then the oxygenated blood is returned to the child’s arterial system at systemic arterial pressure, thus facilitating both oxygenation and systemic vascular output (Fig. 1). The catheters are either placed peripherally (usually via the right common carotid artery) or centrally through a median sternotomy created during surgery (Fig. 2). The former approach requires ligation of the right common carotid artery, which can be later reconstructed after decannulation.

Anticoagulation is required to prevent the formation of thrombi within the ECMO circuit. Together with altered haemodynamics and damage to, or occlusion of, the neck vessels, there is an increased risk of neurologic complications, including intracerebral haemorrhage and ischaemic/embolic infarction [2].

## Relevance of cranial ultrasound in extracorporeal membrane oxygenation support

Cranial US is a safe, portable technique capable of real-time structural assessment and colour phase and spectral Doppler imaging of intracranial arterial and venous flow. It is often used to provide baseline imaging before cardiac surgery, as a bedside monitoring tool after initiation of ECMO support, and for daily follow-up of evolving pathology. If further characterisation of lesions detected on US is required or clinical suspicion persists despite a negative US, CT is the cross-sectional imaging modality of choice because the ECMO equipment is not compatible with the MRI scanner. After ECMO therapy is withdrawn, MRI is the follow-up imaging modality of choice. There are, of course, limitations to cranial US, with the diagnostic quality of cranial US being highly operator-dependent and with reduced visualisation of the posterior fossa and convexity regions. Furthermore, ultrasound has reduced sensitivity and specificity for pathologies other than major haemorrhage compared to both CT and MRI [3–6].

## Technique

**Fig. 3:**
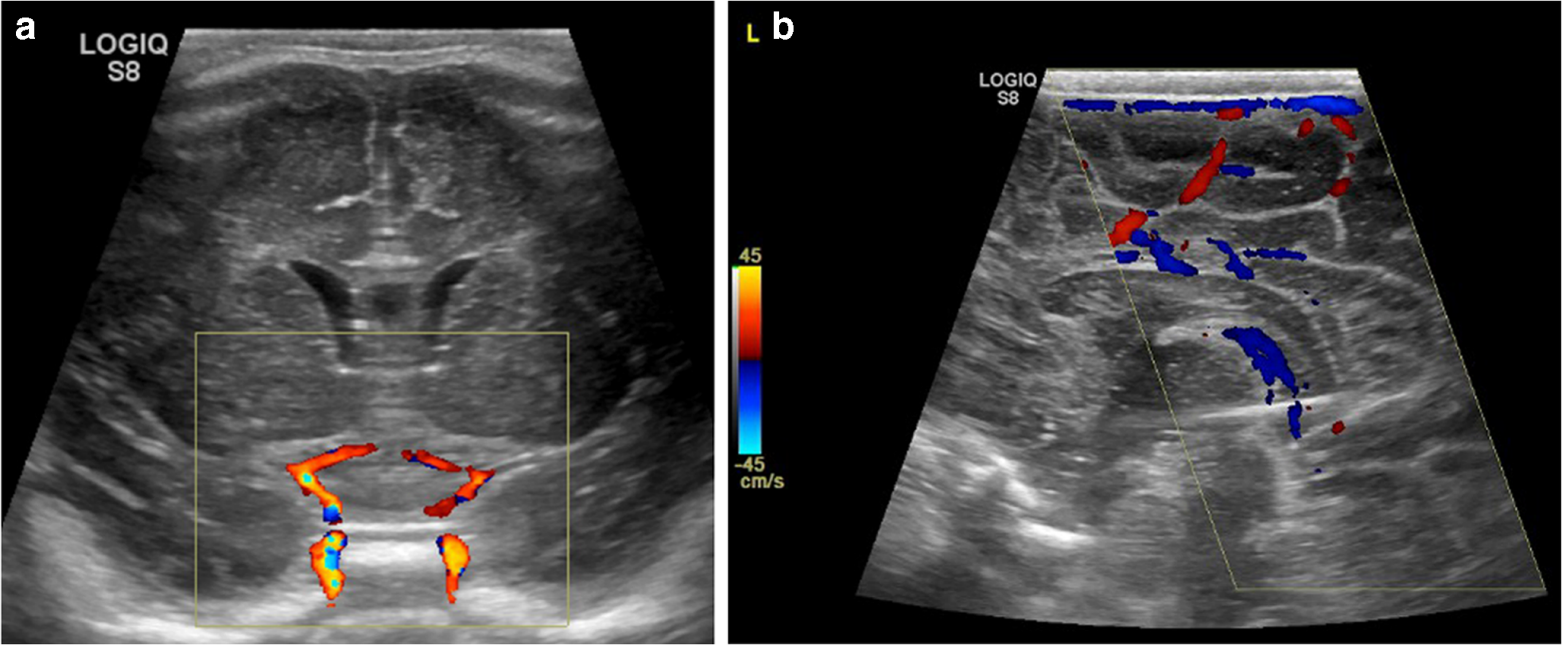
Doppler ultrasound in a 3-day-old boy. **a** In the coronal plane colour Doppler visualises flow in the internal carotid and anterior communicating arteries. **b** In the sagittal plane, the superior sagittal sinus and straight sinus can be visualised

**Fig. 4:**
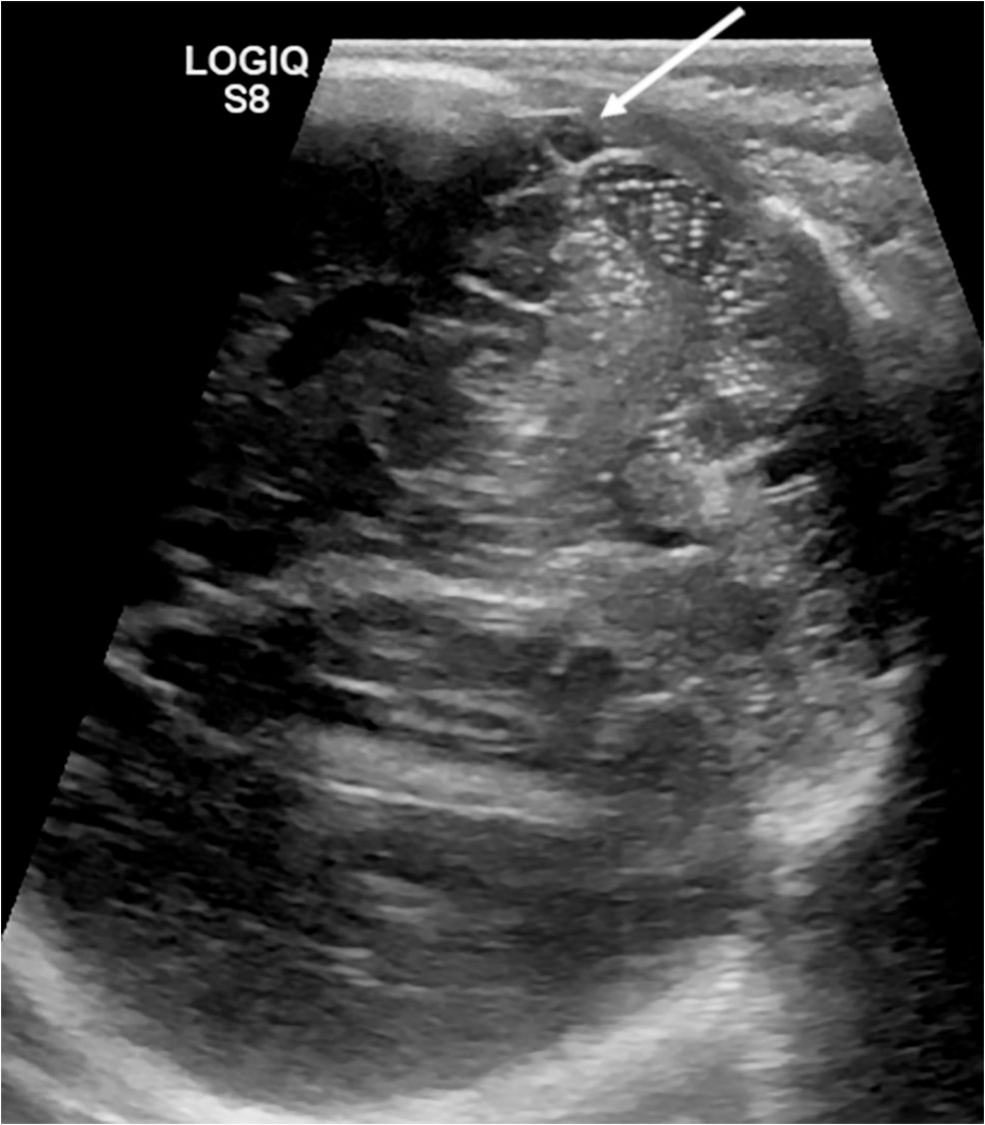
The mastoid fontanelle provides a window (oblique coronal view) to examine the cerebellum and its vermis, the inferior temporal lobes, the midbrain and the cranial portion of the spinal cord in the same 3-day-old boy as in Fig. 3. Much of the transverse venous sinus (*arrow*) can also be demonstrated

The anterior fontanelle provides the largest cranial sonographic window for both coronal and sagittal image acquisition and is usually the last of the fontanelles to close (50% close by 16 months of age) [7]. In addition to grey-scale anatomical images, colour and spectral Doppler assessment can be obtained of the intracranial internal carotid and basilar arteries and the circle of Willis, as well as the superior sagittal sinus, vein of Galen and straight sinus (Fig. 3). Supplemental views of the posterior fossa can be obtained via the posterior and mastoid fontanelles [6] (Fig. 4).

**Fig. 5:**
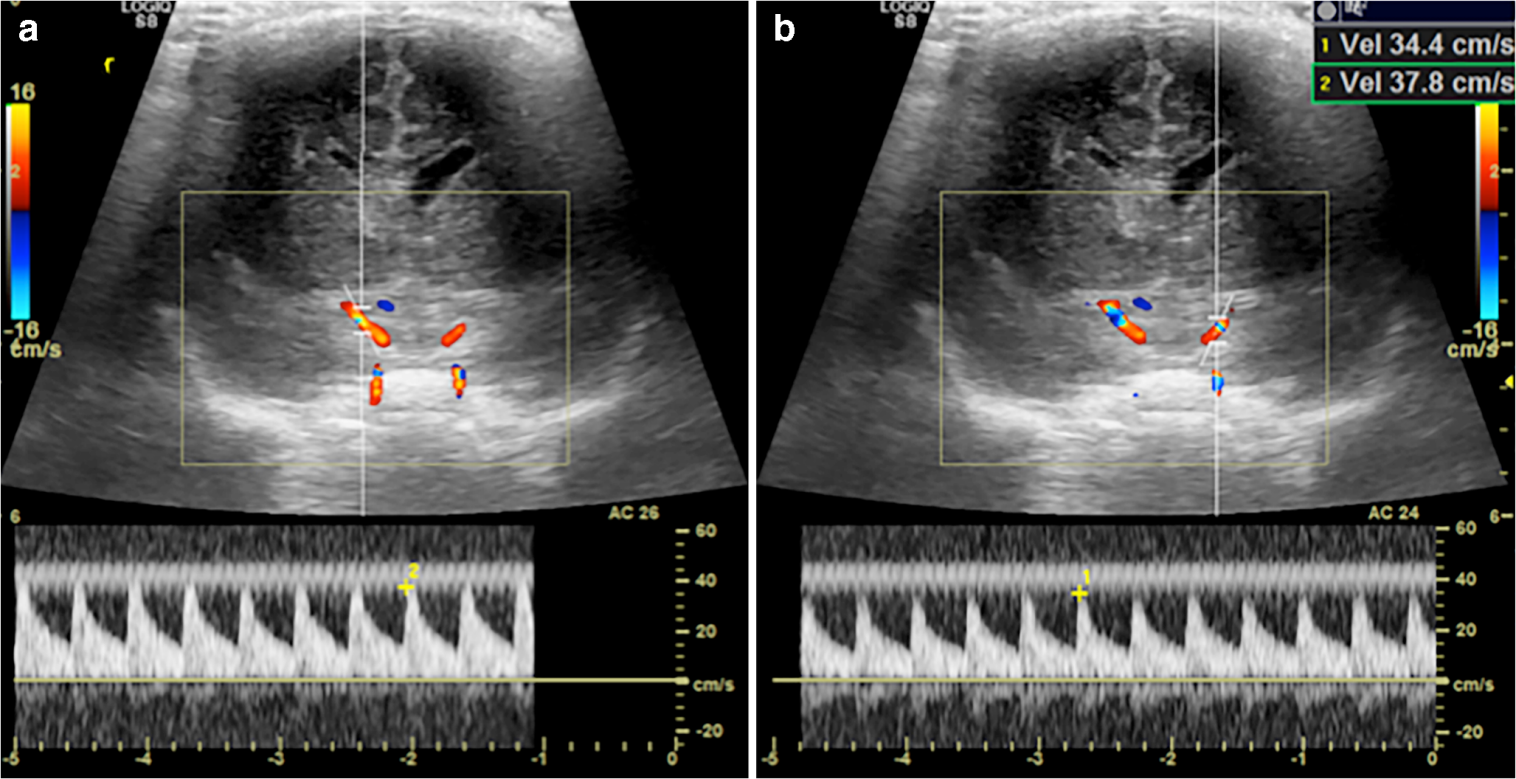
Coronal sonograms in a 1-week-old boy show normal spectral Doppler flow of the internal carotid arteries: low-resistance, pulsatile arterial flow is demonstrated in both intracranial internal carotid arteries with similar peak systolic velocities on each side

**Fig. 6:**
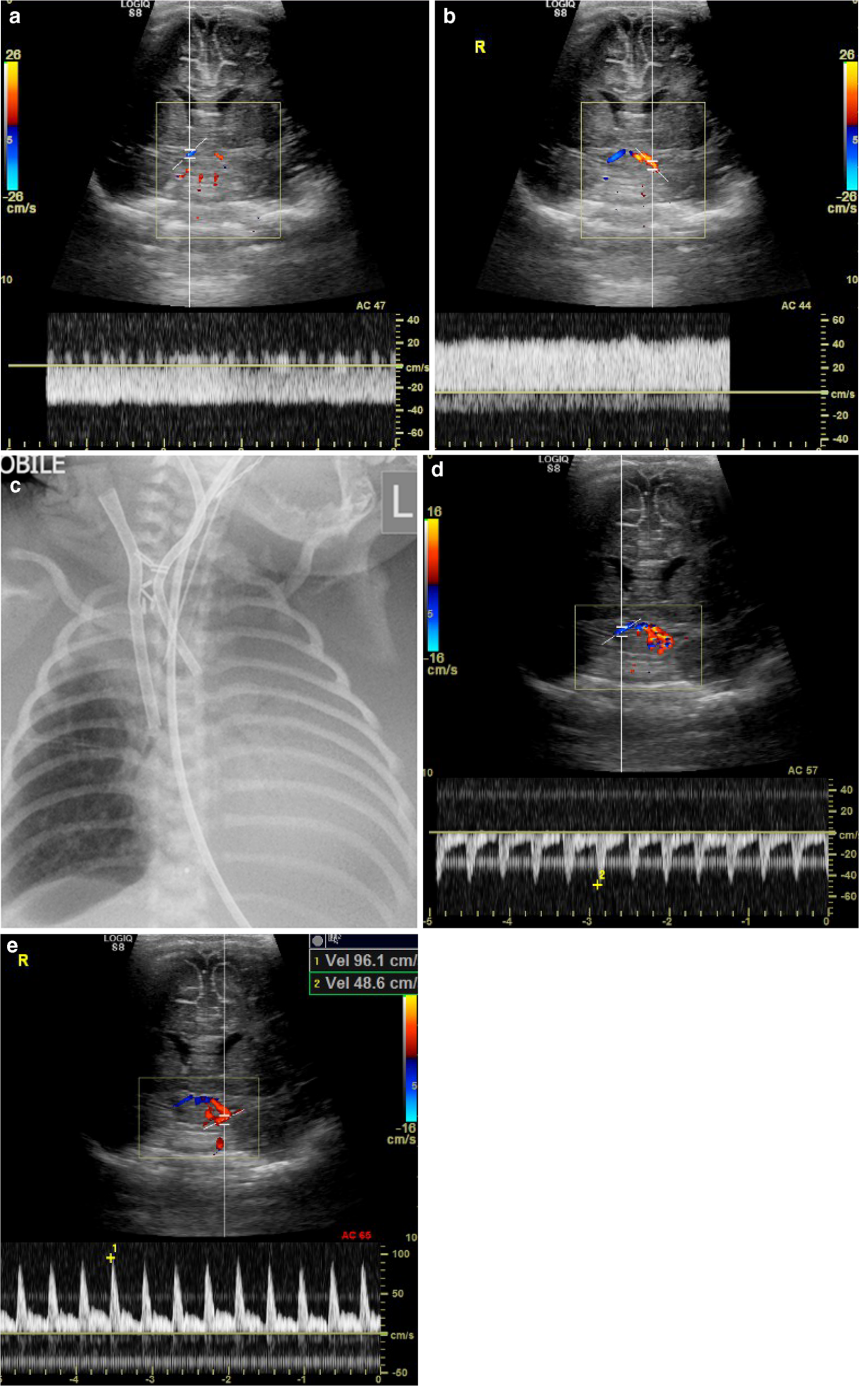
Flow alteration during veno-arterial extracorporeal membrane oxygenation (ECMO) in a 4-day-old boy. **a** Whilst on peripheral veno-arterial ECMO via a ligated right carotid artery, coronal sonogram shows Doppler flow in the intracranial right carotid system is reversed due to supply from the left carotid via the anterior and/or posterior communicating arteries. **b** Coronal sonogram shows Doppler flow in the non-ligated left carotid system remains anterograde. Note the almost absent pulsatility. **c** Anteroposterior chest radiograph of the same child. The right common carotid was ligated for placement of the arterial ECMO cannula, explaining the reversed flow in the intracranial right internal carotid artery (flow is retrograde from the left carotid and basilar via the circle of Willis). **d** After decannulation from ECMO, coronal sonogram with Doppler shows supply to the right intracranial internal carotid system in the same patient remained retrograde (via the anterior communicating artery) implying significant stenosis at the site of common carotid repair. **e** Coronal sonogram shows Doppler flow in the left intracranial internal carotid system remains anterograde and is now pulsatile with a low resistance waveform

In a normal brain, low-resistance waveforms should be evident within both intracranial internal carotid arteries with vertical systolic upstrokes (short acceleration time) and constant forward flow throughout diastole (Fig. 5). In a child on ECMO, ligation of the right common carotid artery results in absent or reversed flow in the right intracranial carotid artery and non-pulsatile forward flow in the other intracranial arteries from the continuous non-pulsatile output of the ECMO pump (Fig. 6). With the right common carotid artery occluded, supply to the right hemisphere occurs via the circle of Willis, with retrograde flow through the right posterior or anterior communicating arteries. This retrograde flow might persist after decannulation from extracorporeal membrane oxygenation support, even following surgical reconstruction of the common carotid artery at decannulation [8] (Fig. 6d and e). Reassuringly, even in cases of persistent internal carotid stenosis post reconstruction, the vast majority of children retain symmetrical cerebral perfusion, in keeping with good collateralisation and compensation via the circle of Willis [9, 10].

## Abnormal findings

### Haemorrhage

Intracranial haemorrhage is the most frequent neurologic complication in neonates receiving ECMO support [11], the majority of cases occurring within 72 h of initiation of ECMO support [6]. It is notable that most non-survivors of ECMO support die from intracranial haemorrhage rather than their primary cardiorespiratory disease [2].

**Fig. 7:**
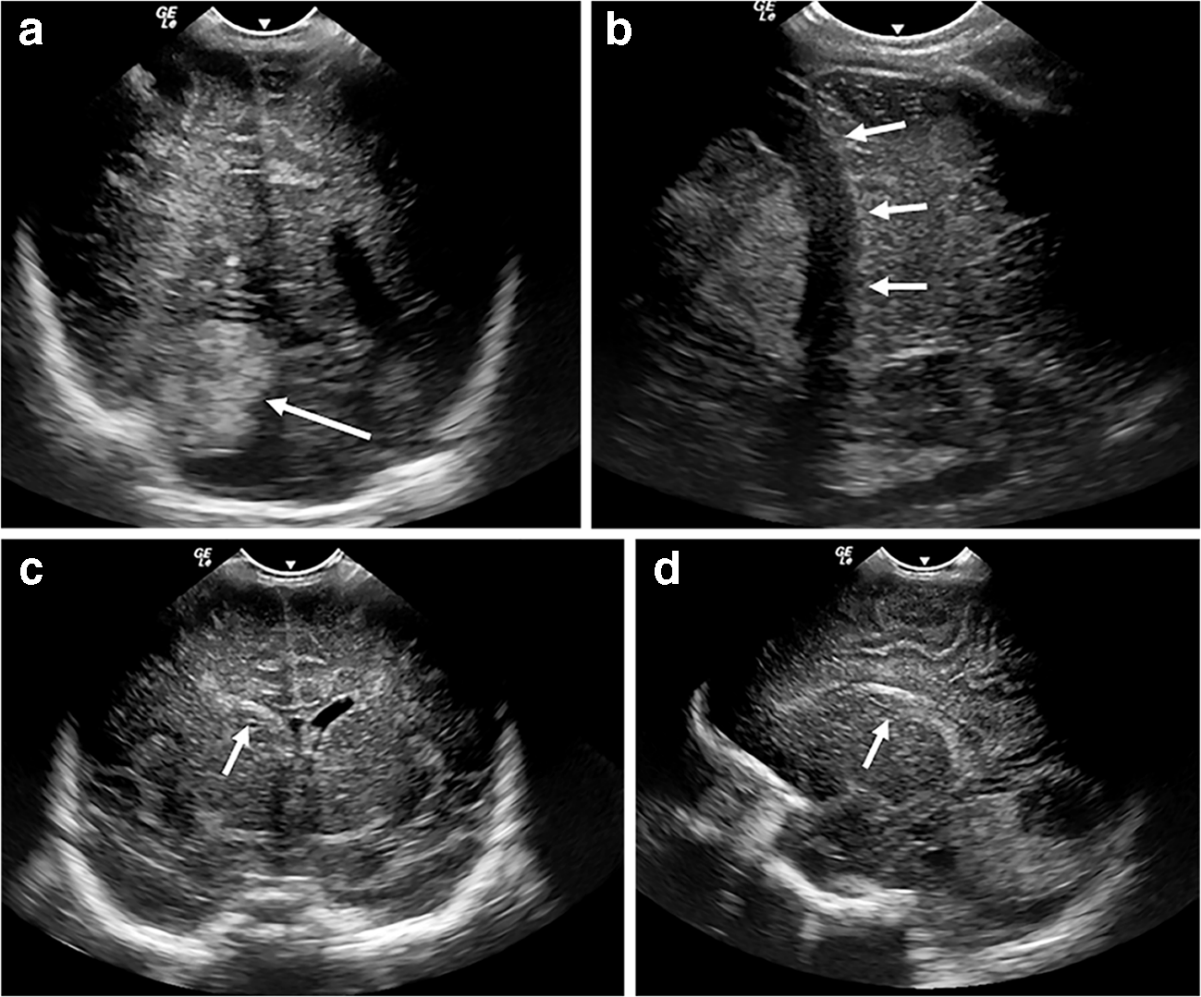
Intraparenchymal, subdural and intraventricular haemorrhage on sonography in a 1-day-old boy. Continuous anticoagulation is generally administered while a child is on extracorporeal membrane oxygenation therapy to prevent thrombus forming within the circuit. These children are therefore at increased risk of spontaneous intracranial haemorrhage. **a** Coronal sonogram shows echogenic material in the right parieto-occipital region, in keeping with a parenchymal haematoma (*arrow*). **b** Anechoic fluid (*arrows*) within the adjacent subdural space, demonstrated via an oblique coronal view from the mastoid fontanelle, in keeping with acute subdural extension. **c**, **d** Blood (*arrows*) is also demonstrated within the right lateral ventricle in the coronal (**c**) and sagittal (**d**) planes with mild mid-line shift evident.

**Fig. 8:**
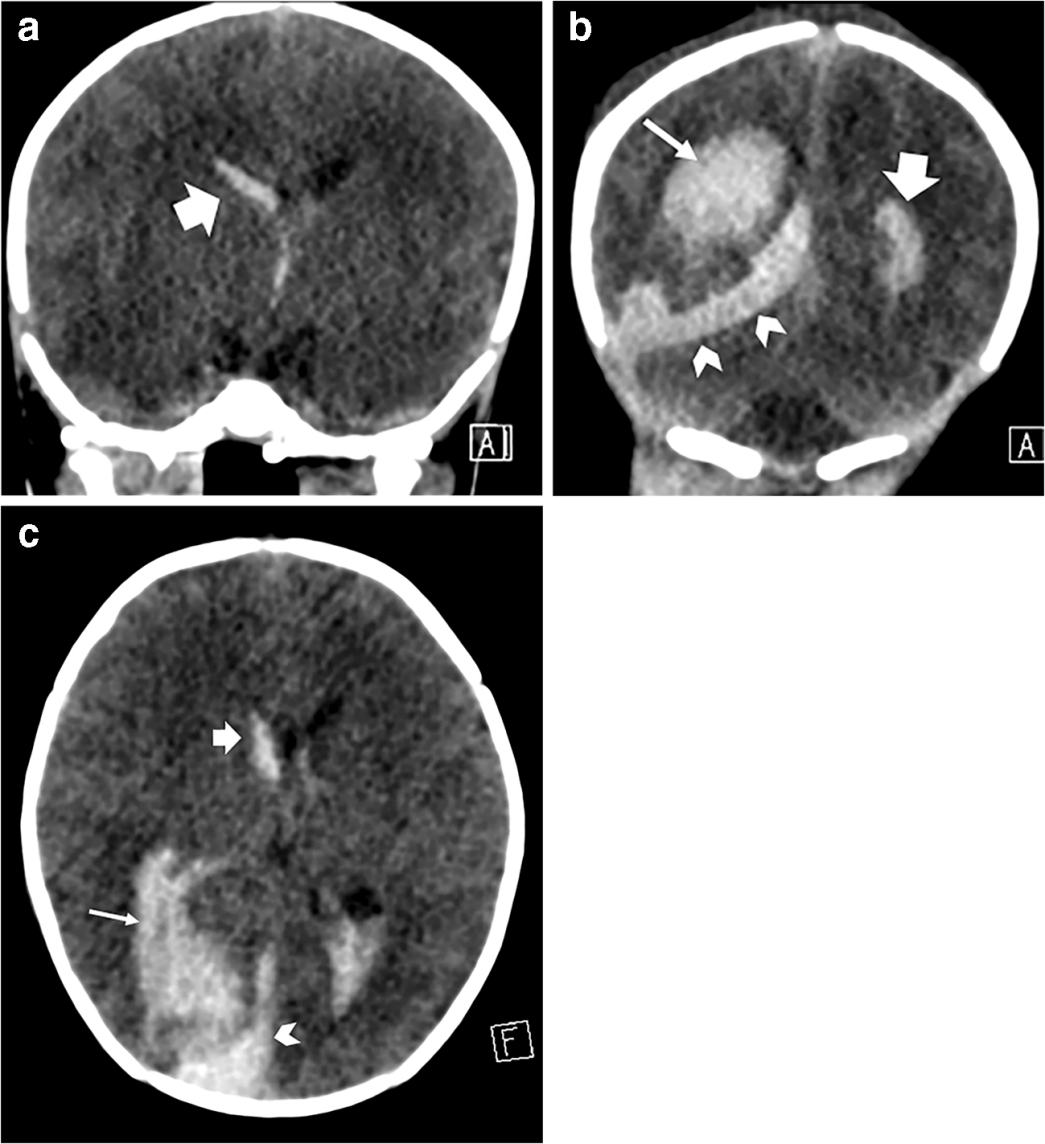
Intraparenchymal, subdural and intraventricular haemorrhage on CT in the same 1-day-old boy as in Fig. 7. Coronal sections through the third ventricle (**a**) and posterior horns (**b**), and axial (**c**) CT image confirm a right parieto-occipital haematoma (*thin arrow*) with subdural (*chevrons*) and intraventricular (*thick arrow*) extension. CT better demonstrates the extent of haemorrhage and any resulting midline shift and is used as a confirmatory test following ultrasound or when clinical suspicion persists despite a negative cranial US examination

Intracranial sites of haemorrhage visible on ultrasound include the germinal matrix, ventricular system, cerebral and cerebellar parenchyma and extra-axial spaces (subarachnoid, subdural and extradural spaces). On ultrasound, haematoma normally appears as echogenic (bright) space-occupying material (Fig. 7), and depending on the volume and location of haemorrhage, it might be accompanied by midline shift or dilatation of the lateral ventricles. If patient transfer is safe, the extent of haemorrhage should ideally be confirmed via subsequent CT, which shows haemorrhage as hyperattenuating material (Fig. 8).

### Hydrocephalus

**Fig. 9:**
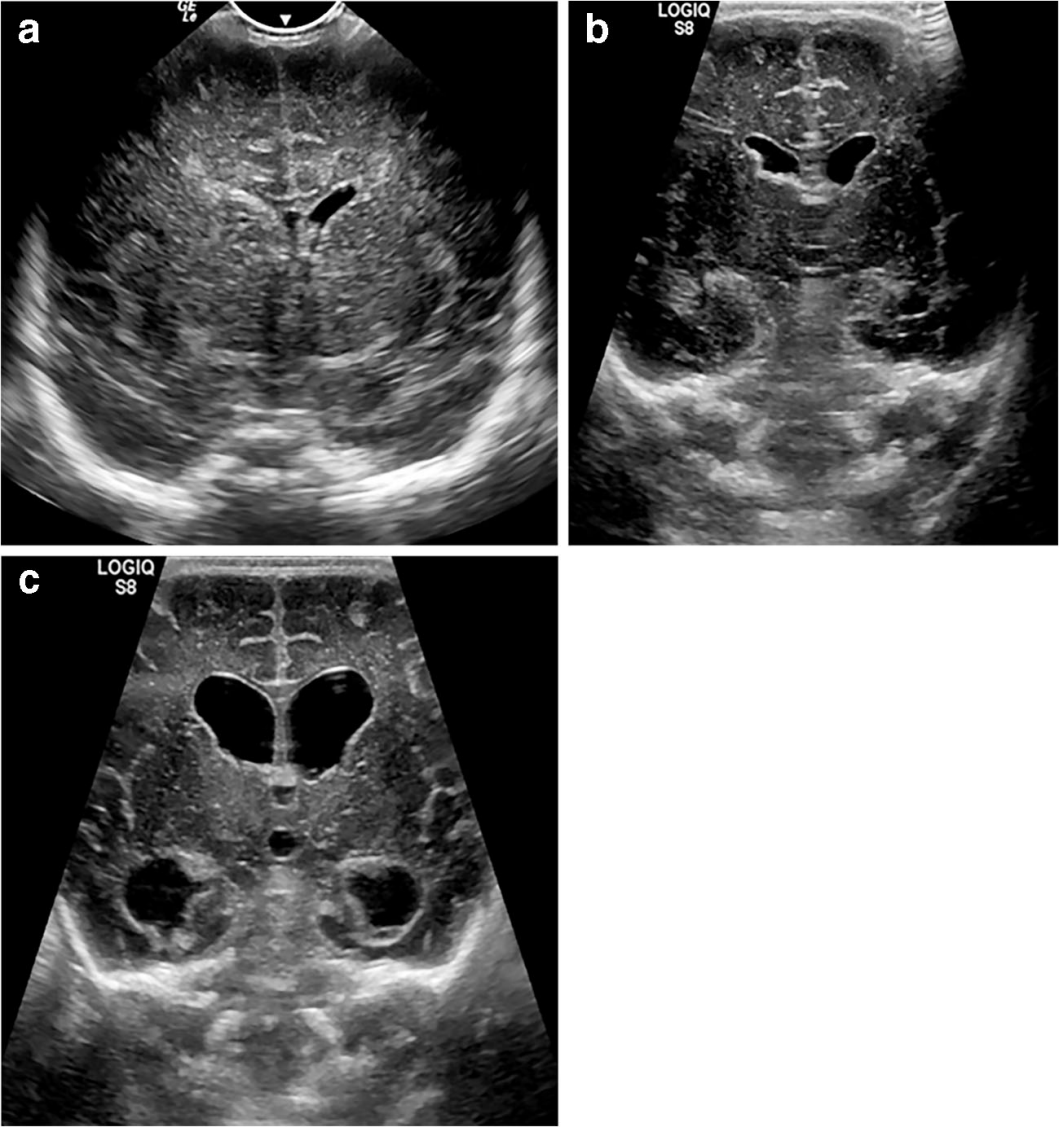
Evolution of hydrocephalus on cranial US. Coronal sonograms in the same 1-day-old boy shown in Figs. 7 and 8. **a** At the time of the acute haemorrhage with haematoma in the right lateral ventricle. **b** At 48 h. **c** At 72 h. There is progressive ventricular dilatation, in keeping with communicating hydrocephalus. Ultrasound provides a repeatable, ionising-radiation-free follow-up test that can be performed at the child’s bedside

Intraventricular haemorrhage can obstruct arachnoid granulation tissue, resulting in communicating hydrocephalus (Fig. 9), at times requiring neurosurgical intervention. The degree of hydrocephalus can be monitored via serial ultrasonography with measurement of the ventricular index (e.g., Levene index) or dimensions/area of the lateral ventricular horns.

### Evolving haematoma

**Fig. 10:**
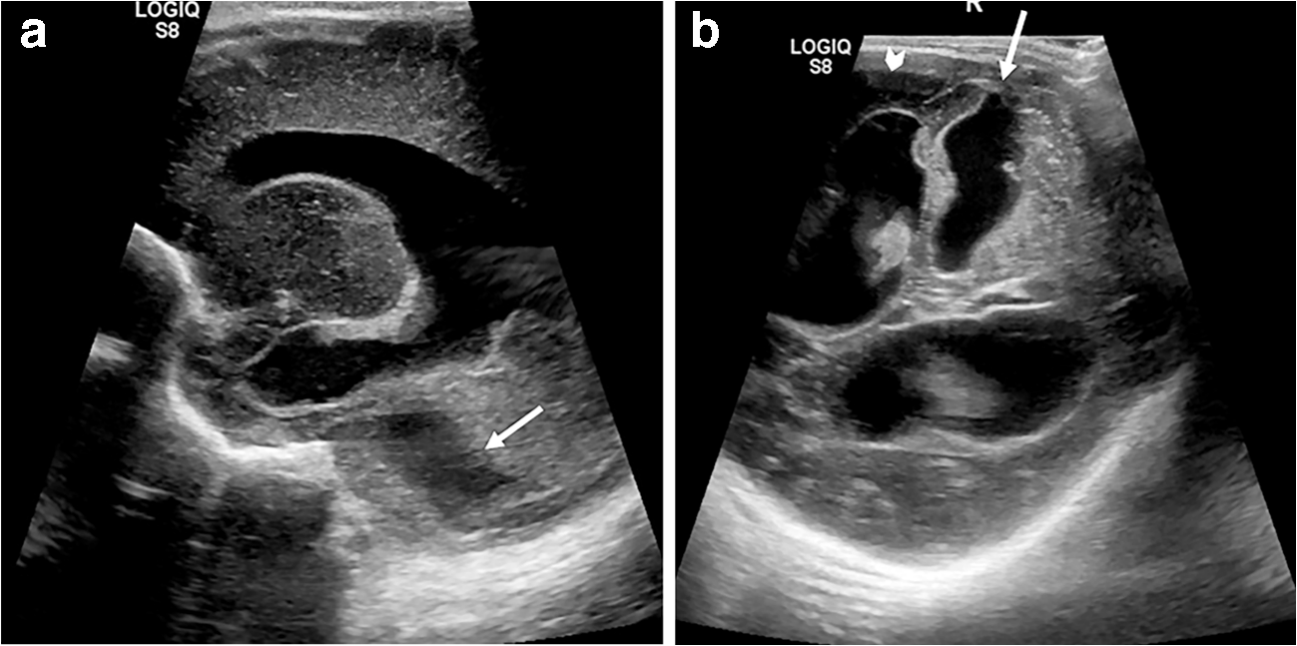
Evolution of haematoma on cranial US in the same boy as in Figs. 7, 8 and 9 at 3 weeks old. **a**, **b** Sagittal view (**a**) and oblique coronal view via the mastoid/lambdoid fontanelle (**b**) views demonstrate anechoic fluid (*arrow*) in the region of the previously demonstrated hypoechoic subdural haematoma, in keeping with liquefied haematoma. The cerebral parenchyma at the site of the parenchymal bleed is thinned (*chevron* in **b**) and subsequently formed a porencephalic cyst

As haematoma begins to liquefy, particularly in the extra-axial spaces, it can become hypo- or even anechoic, often containing echogenic debris or even a debris–fluid level (Fig. 10). Serial ultrasound examinations can track the evolution of previously confirmed haematoma without the need for repeated CT or MRI examinations.

### Ischaemia

**Fig. 11:**
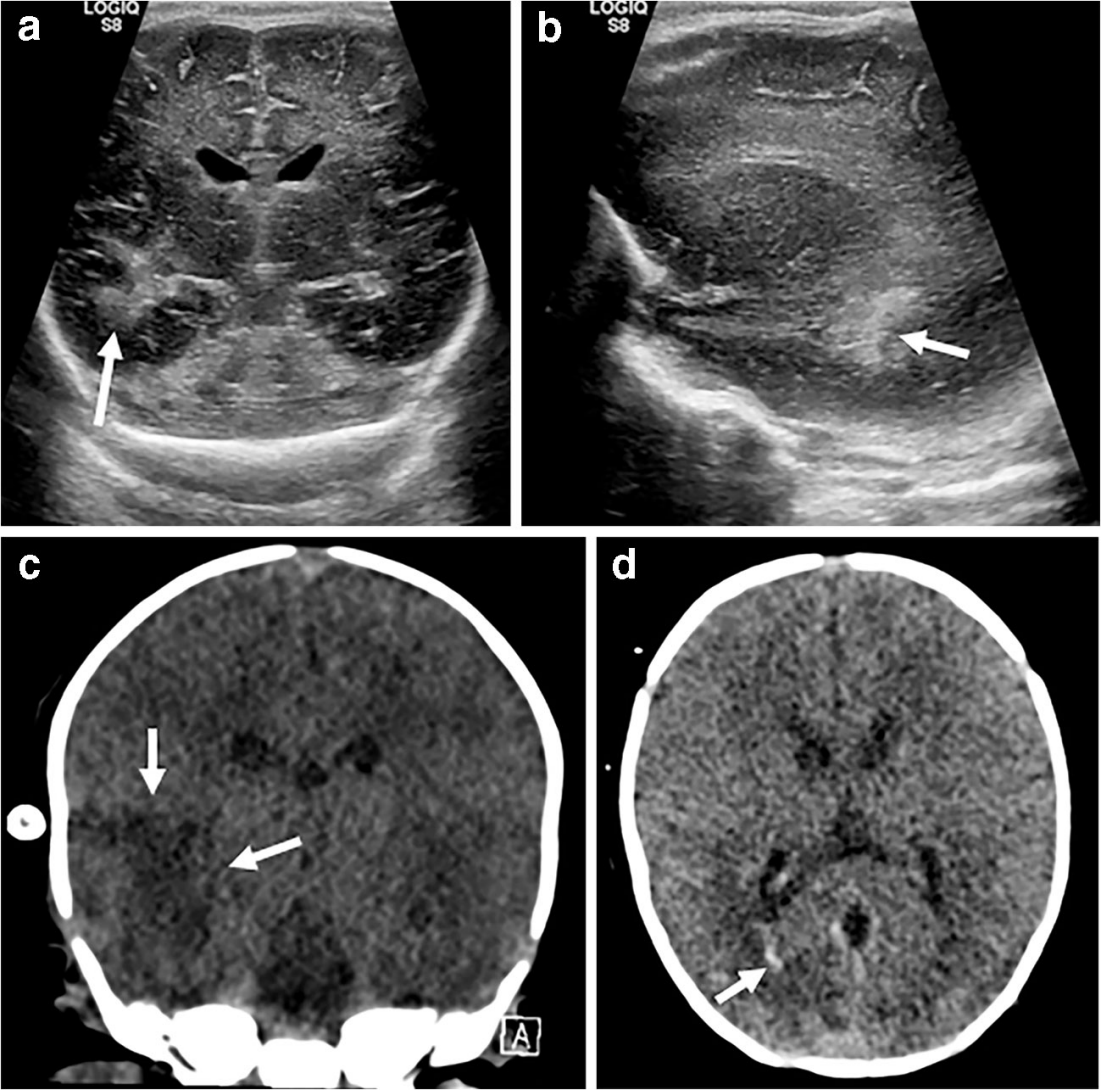
Arterial infarct with haemorrhagic transformation on cranial US and CT in an 8-day-old boy. **a, b** Coronal (**a**) and parasagittal (**b**) ultrasound images demonstrate a hyperechoic focus in the right temporal lobe (*arrow*), consistent with a focal infarct. **c, d** CT in the coronal (**c**) and axial (**d**) planes confirms the infarct (*arrows* in **c**) and additionally demonstrates acute blood in the trigone of the right lateral ventricle (*arrow* in **d**). Arterial ischaemia is relatively common in this group of patients — in addition to increased risk from cardiac shunts and surgical procedures, extracorporeal membrane oxygenation therapy carries a risk of thrombus formation and embolism, risk of increased bleeding from heparinisation, as well as the potential for insufficient collateral flow via the circle of Willis after ligation of the right common carotid artery. Established ischaemic infarcts appear hyperechoic on ultrasound, often with loss of overlying grey matter. Whilst a large territorial infarct can be convincingly demonstrated by ultrasound, CT is still advised to confirm the extent and assess for small associated areas of haemorrhage that might be impossible to demonstrate on ultrasound. It is also worth mentioning that hypoattenuation often develops sooner on CT than hyperechogenicity appears on ultrasound. If a cranial ultrasound appears normal, but clinical suspicion of ischaemic infarct is high, further imaging (CT or MRI as appropriate/possible) is still indicated

After haemorrhage, the second most common major neurologic injury sustained by infants on extracorporeal membrane oxygenation is ischaemia, whether secondary to underlying pathology (e.g., complex cyanotic congenital heart disease), complications of surgery or initiation of ECMO support (emboli, ligation of the common carotid artery with insufficient collateral flow) or a combination of factors. On ultrasound, regions of established ischaemic infarction appear hyperechoic, with loss of grey–white matter differentiation occasionally appreciable on high-frequency imaging. Infarcts often occupy characteristic vascular territories but are occasionally more localised (either as small wedge-shape infarcts or focal lacunar infarcts). If small and localised they can be difficult to distinguish from focal haemorrhage, in which case correlation with cross-sectional imaging (CT if continued extracorporeal membrane oxygenation support is required) is useful (Fig. 11).

**Fig. 12:**
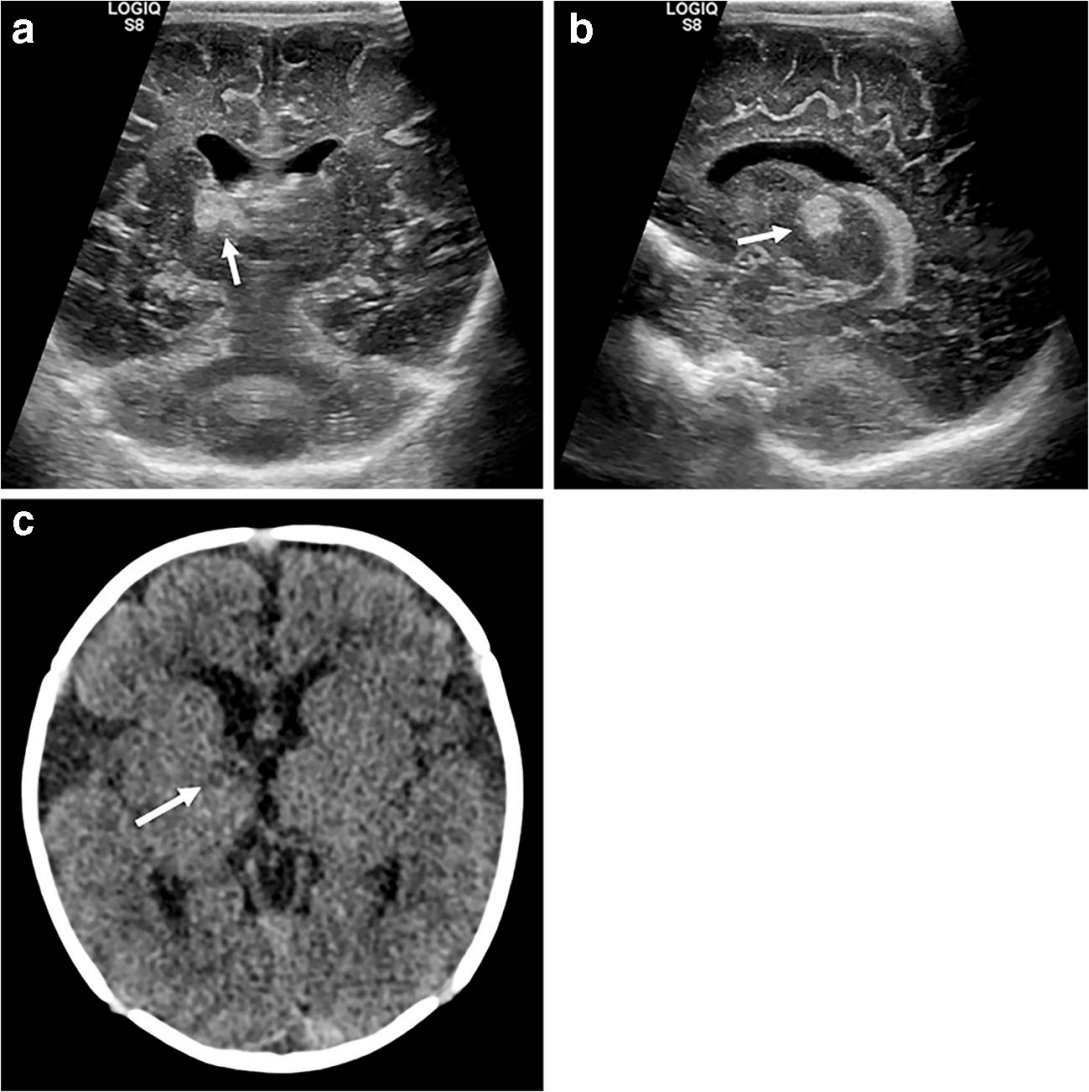
Venous infarct on cranial US and CT in a 1-month-old girl. **a**, **b** Coronal (**a**) and sagittal (**b**) sonograms show a focal hyperechogenicity (*arrow*) in the right thalamus, initially thought to be a haematoma. **c** Axial CT, however, shows that the lesion (*arrow*) is an infarct. Venous infarcts occur secondary to central venous thrombosis and should be suspected if the stroke appears to be in a non-arterial vascular territory, typically affecting parasagittal structures. Venous infarcts are often complicated by haemorrhage and, again, CT is a useful tool for distinguishing them clarification.

Venous infarcts typically occur in a non-arterial distribution and are most frequently visible as hyperechoic regions in the thalami or basal ganglia (Fig. 12). Infarcts can undergo haemorrhagic transformation, with the echogenic appearance of both infarct and haemorrhage on ultrasound, leading to potential confusion. In cases of diagnostic uncertainty, further interrogation with CT is helpful.

### Cortical laminar necrosis

**Fig. 13:**
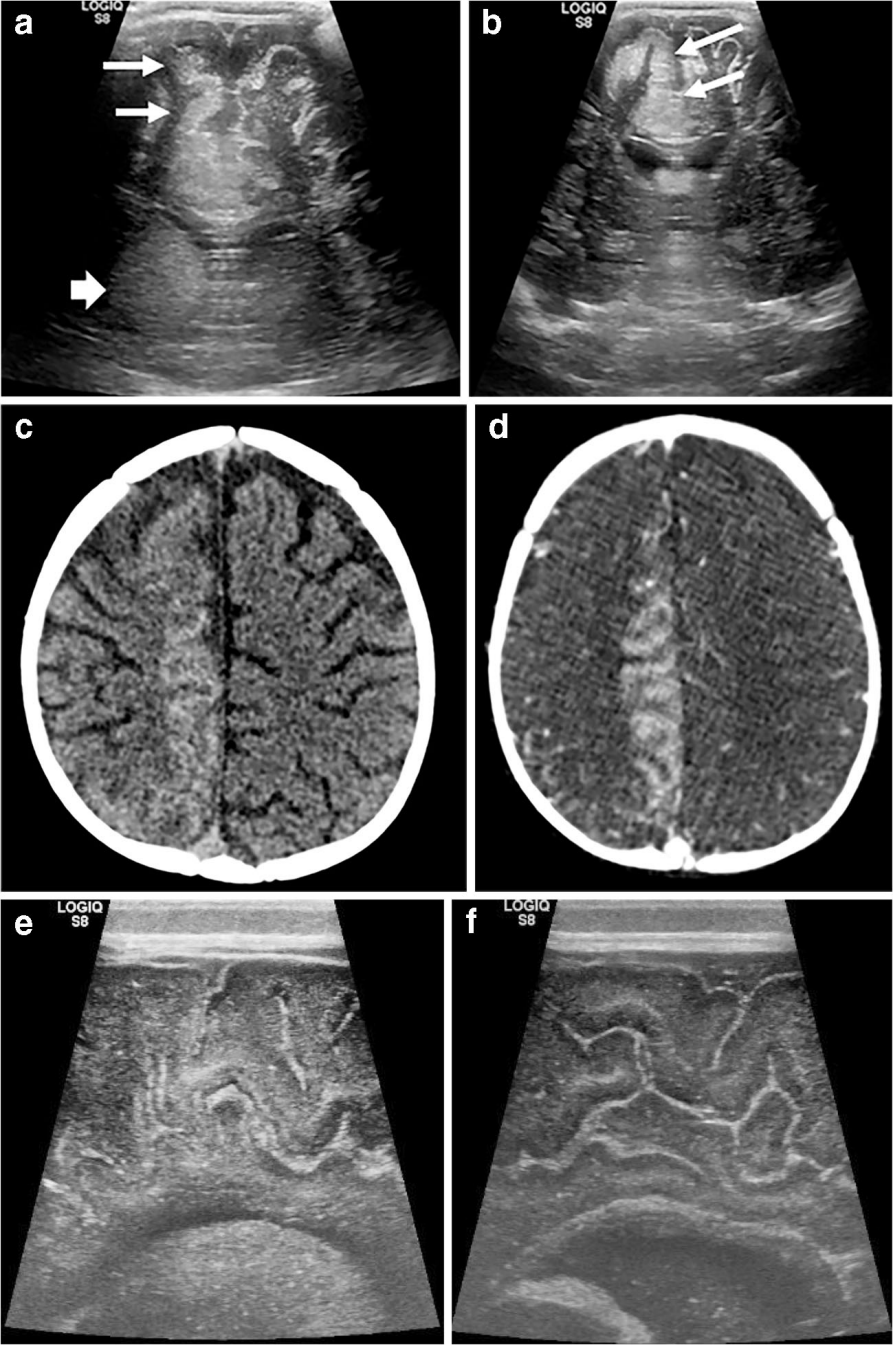
Cortical laminar necrosis on cranial US, CT and contrast-enhanced CT in a 5-month-old girl. **a**, **b** Coronal US images demonstrate thickened echogenic cortex in the right frontal lobe (*thin arrows*) and hyperechoic right caudate (*thick arrow* in **a**) in an infant recently decannulated from extracorporeal membrane oxygenation following Stage I Norwood procedure for hypoplastic left heart syndrome. **c** Unenhanced axial CT demonstrates hyperattenuating right parasagittal cortex, in keeping with laminar necrosis. **d** Axial CT following the administration of contrast medium shows corresponding enhancement. **e, f** High-frequency sagittal sonograms show loss of grey–white matter differentiation in the right parasagittal cortex (**e**) compared to the normal left parasagittal cortex (**f**). Cortical laminar necrosis is the death of cortical neurons caused by deprivation of oxygen and glucose. This can occur secondary to generalised hypoperfusion or hypoxia and presents radiologically with cortical oedema with loss of grey–white matter differentiation. Whilst laminar necrosis appears bright on CT and T1-weighted MRI, susceptibility-weighted MRI studies have shown that haemorrhage is not often present. It has been suggested that the appearance is secondary to proteinaceous debris rather than blood products

Cerebral energy depletion, described in conditions including infarction and prolonged hypoxic–ischaemic encephalopathy, can lead to necrosis of cortical lamina. This can be associated with diffuse, focal or punctate cortical haemorrhage and can later calcify [1]. The appearance on ultrasound is not well described, but is likely to vary according to the presence and extent of necrosis. Unenhanced CT might demonstrate high-attenuation cortex with further cortical hyperenhancement following contrast administration (Fig. 13). Unenhanced MRI demonstrates high cortical T1 signal. Recent studies have shown that the high attenuation on CT and corresponding high T1 signal on MRI only rarely represent haemorrhage, as once thought, and likely relate to protein degradation within necrotic grey matter [12].

### Periventricular leukomalacia

**Fig. 14:**
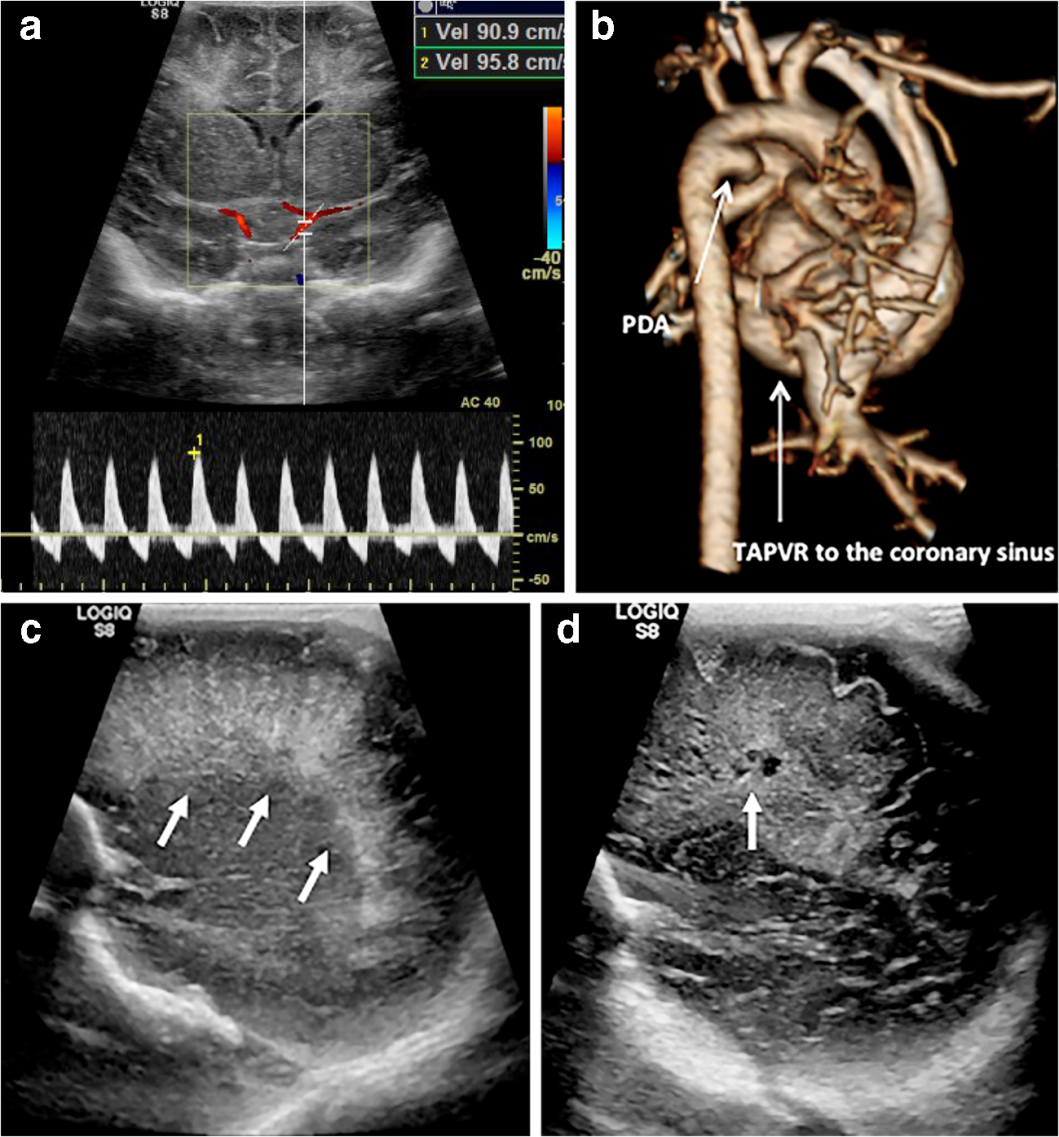
Cystic periventricular leukomalacia in a 3-day-old boy. **a** Coronal cranial US with spectral Doppler demonstrates marked diastolic flow reversal in a neonate. **b** Volume-rendered CT viewed from right shows a large patent ductus arteriosus (*PDA*) and total anomalous pulmonary venous return (*TAPVR*) resulting in a significant steal phenomenon. **c** Parasagittal sonography shows marked periventricular white matter hyperechogenicity (*arrows*) concerning for developing periventricular leukomalacia. Operative management of the congenital heart disease was delayed by systemic sepsis. **d** Subsequent parasagittal sonogram showed that the infant had developed cystic periventricular leukomalacia (*arrow*). Periventricular leukomalacia occurs secondary to watershed ischaemia and presents as a spectrum from mild reversible periventricular white matter hyperechogenicity to frank cystic change with subsequent parenchymal volume loss

Periventricular leukomalacia is a white matter injury related to watershed ischemia of the immature cerebrovascular system, frequently seen in premature neonates. There are two different forms: diffuse and cystic. The diffuse form can be more difficult to detect on ultrasound and might require evaluation with MRI [3]. The cystic form initially appears as increased periventricular white matter echogenicity, and later develops into multiple anechoic periventricular cysts (Fig. 14). It is important from a prognostication point because cystic periventricular leukomalacia is associated with a worse neurologic outcome [13].

### Lenticulostriate vasculopathy

**Fig. 15:**
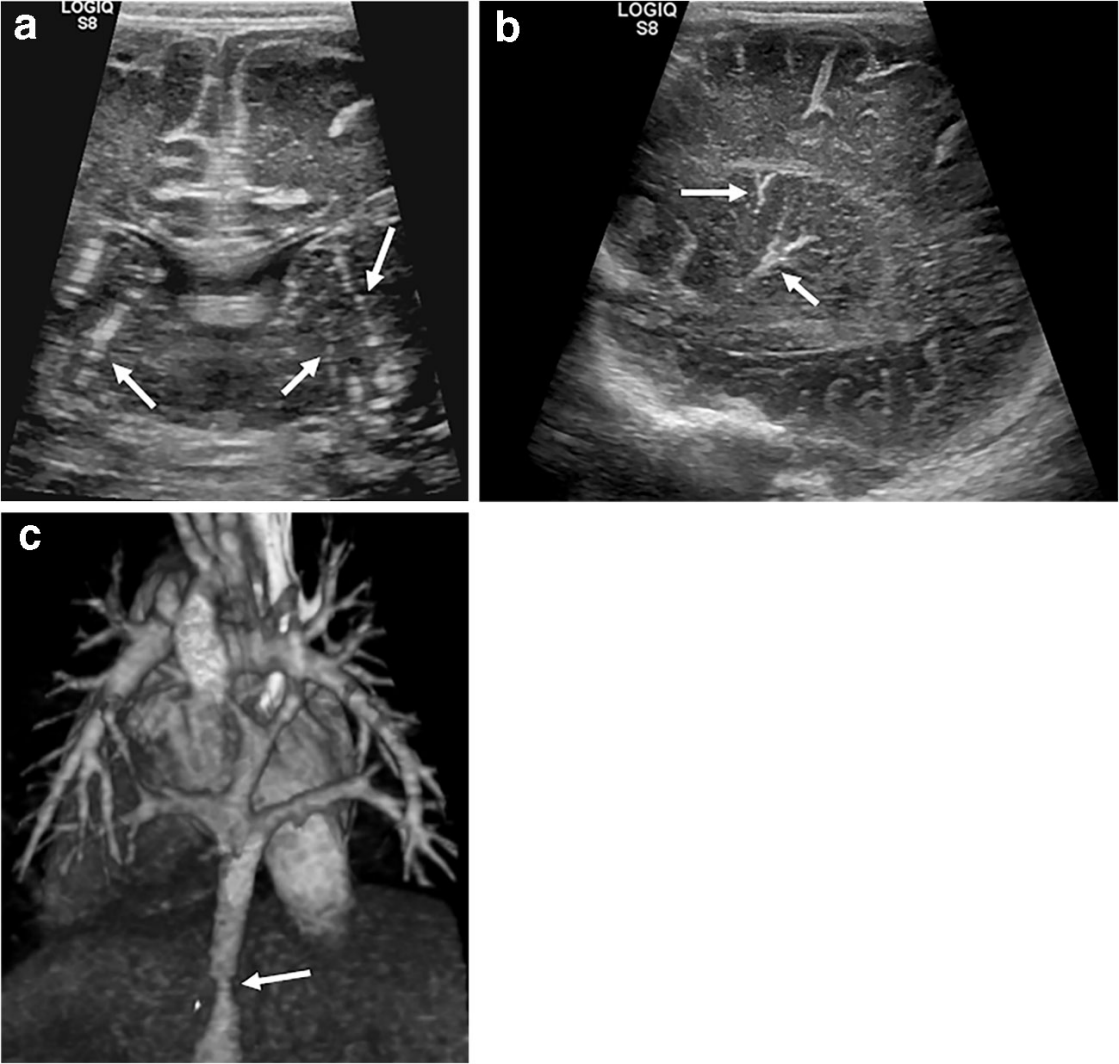
Lenticulostriate vasculopathy on cranial US in a 2-day-old girl. **a, b** Magnified coronal (**a**) and sagittal (**b**) ultrasound images demonstrate echogenic branching vessels (*arrows*) in the region of the basal ganglia. **c** The child had obstructed total anomalous pulmonary venous return as shown on volume-rendered cardiac CT viewed from the posterior with the aorta cut away. The stenosisis of the vertical vein is marked (*arrow*). Lenticulostriate vasculopathy is associated with neonatal infections (TORCH [toxoplasmosis, other, rubella, cytomegalovirus and herpes] infections), chromosomal anomalies (particularly common in Trisomy 21) and hypoxia

Lenticulostriate vasculopathy is thought to be caused by thickening of the lenticulostriate branches of the middle cerebral arteries and leads to branching echogenic structures becoming visible within the basal ganglia on cranial US. The specific pathogenesis is not fully understood [14], although it is associated with a number of underlying disorders including congenital heart disease and hypoxic–ischaemic events [15]. Unsurprisingly, it is therefore frequently seen in infants on ECMO support (Fig. 15).

### Diffuse hypoxic–ischaemic encephalopathy

**Fig. 16:**
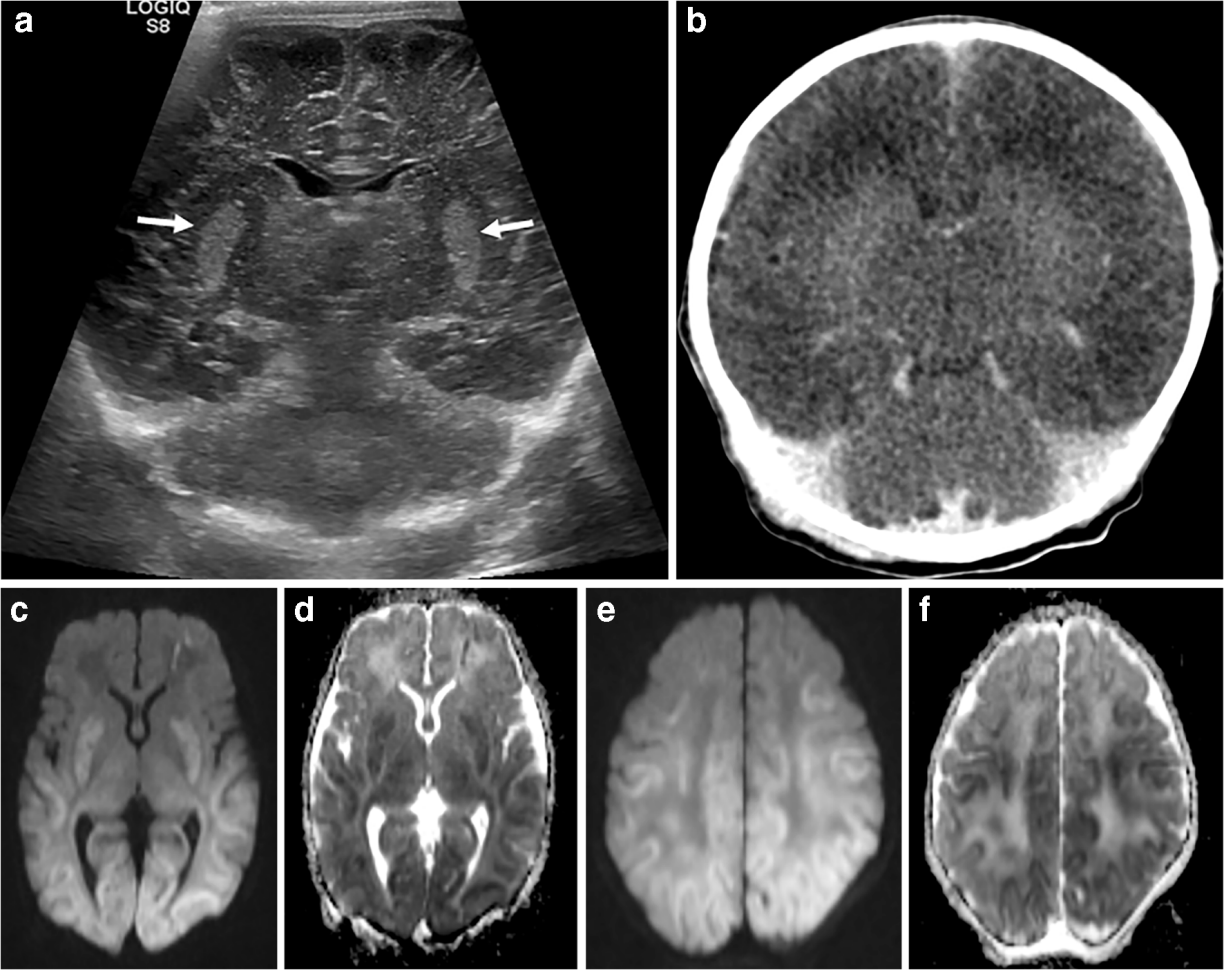
Diffuse hypoxic–ischaemic encephalopathy in a 2-week-old boy with congenital cardiac disease, following prolonged cardiac arrest. **a** Coronal sonogram shows echogenic basal ganglia (*arrows*). **b** Coronal CT demonstrates diffuse low-attenuation and effacement of the lateral ventricles, in keeping with diffuse ischaemic injury and diffuse cerebral oedema (there is residual intravenous contrast material from preceding cardiac catheterisation). **c**–**f** Axial sections of matched b=1,000 diffusion-weighted MR images (**c**, **e**) and apparent diffusion coefficient maps (**d**, **f**) through the lateral ventricles (**c**, **d**) and white matter (**e**, **f**) demonstrate diffusion restriction in the basal ganglia and posterior cortex, in keeping with acute ischaemia. The deep grey matter is highly metabolically active and, as a result, is the first region to be affected by hypoperfusion. Ultrasound changes can initially be extremely subtle, but the combination of a significant history (often a cardiac arrest with prolonged cardiopulmonary resuscitation) and increased basal ganglia echogenicity should prompt further imaging when safe and practicable

Severe diffuse hypoxic–ischaemic encephalopathy in neonates initially results in a central pattern of damage, involving deep grey matter (basal ganglia and thalami), but with prolonged ischaemia, the cortex can also become involved. Ultrasound might initially appear normal or demonstrate a mild global increase in cerebral echogenicity with or without diffuse swelling obliterating the cerebrospinal fluid spaces. Increased echogenicity of the thalami (Fig. 16) suggests more severe injury and is associated with a poorer outcome [14]. Doppler examination of the anterior and middle cerebral arteries has been employed in the setting of suspected hypoxic–ischaemic encephalopathy, with a resistive index of less than 0.6 associated with poor outcome, even if other sonographic signs are absent [16].

## Conclusion

Cranial US is a safe, portable technique allowing screening prior to, and monitoring whilst on, extracorporeal membrane oxygenation support. Whilst ultrasound is the first-line imaging technique in these children, significant limitations (particularly in the setting of suspected acute ischaemia) leave CT as the modality of choice for confirming and demonstrating the extent of significant pathologies whilst a child is on ECMO support, particularly when important treatment decisions are required, often including the decision as to whether to continue ECMO support.

## References

[1] Raets MMA, Dudink J, Ijsselstijn H (2013). Brain injury associated with neonatal extracorporeal membrane oxygenation in the Netherlands: a nationwide evaluation spanning two decades. Pediatr Crit Care Med.

[2] de Mol AC, Liem KD, van Heijst AFJ (2013). Cerebral aspects of neonatal extracorporeal membrane oxygenation: a review. Neonatology.

[3] van Wezel-Meijler G, Steggerda SJ, Leijser LM (2010). Cranial ultrasonography in neonates: role and limitations. Semin Perinatol.

[4] Rollins MD, Yoder BA, Moore KR (2012). Utility of neuroradiographic imaging in predicting outcomes after neonatal extracorporeal membrane oxygenation. J Pediatr Surg.

[5] LaRovere KL, Vonberg FW, Prabhu SP (2017). Patterns of head computed tomography abnormalities during pediatric extracorporeal membrane oxygenation and association with outcomes. Pediatr Neurol.

[6] van Heijst AFJ, de Mol AC, Ijsselstijn H (2014). ECMO in neonates: neuroimaging findings and outcome. Semin Perinatol.

[7] Pindrik J, Ye X, Ji BG (2014). Anterior Fontanelle closure and size in full-term children based on head computed tomography. Clin Pediatr.

[8] Di Salvo DN (2001). A new view of the neonatal brain: clinical utility of supplemental neurologic US imaging windows. Radiographics.

[9] Mitchell DG, Merton D, Desai H et al (1988) Neonatal brain: color Doppler imaging. Part II. Altered flow patterns from extracorporeal membrane oxygenation. Radiology 167:307–310 10.1148/radiology.167.2.3282251 3282251

[10] Wien MA, Whitehead MT, Bulas D (2017). Patterns of brain injury in newborns treated with extracorporeal membrane oxygenation. AJNR Am J Neuroradiol.

[11] Lago P, Rebsamen S, Clancy RR (1995). MRI, MRA, and neurodevelopmental outcome following neonatal ECMO. Pediatr Neurol.

[12] Niwa T, Aida N, Shishikura A (2008). Susceptibility-weighted imaging findings of cortical laminar necrosis in pediatric patients. AJNR Am J Neuroradiol.

[13] Murgo S, Avni EF, David P (1999). Periventricular leukomalacia in premature infants: prognostic role of ultrasonography and MRI. J Radiol.

[14] Shin HJ, Kim M-J, Lee HS (2015). Imaging patterns of sonographic lenticulostriate vasculopathy and correlation with clinical and neurodevelopmental outcome. J Clin Ultrasound.

[15] Lowe LH, Bailey Z (2011). State-of-the-art cranial sonography: part 2, pitfalls and variants. AJR Am J Roentgenol.

[16] Stark JE, Seibert JJ (1994). Cerebral artery Doppler ultrasonography for prediction of outcome after perinatal asphyxia. J Ultrasound Med.

